# Chemo- and Diastereoselectivities in the Electrochemical Reduction of Maleimides

**DOI:** 10.1002/cssc.201403184

**Published:** 2015-01-08

**Authors:** Kathryn Rix, Geoffrey H Kelsall, Klaus Hellgardt, King Kuok (Mimi) Hii

**Affiliations:** [a] Department of Chemistry, Imperial College London Exhibition Road, South Kensington, London SW7 2AZ (UK) E-mail: mimi.hii@imperial.ac.uk; [b] Department of Chemical Engineering, South Kensington Campus, Imperial College London London SW7 2AZ (UK)

**Keywords:** chemoselectivity, diastereoselectivity, electrochemistry, reaction mechanisms, reduction

## Abstract

The electrochemical cathodic reduction of cyclic imides (maleimides) to succinimides can be achieved chemoselectively in the presence of alkene, alkyne, and benzyl groups. The efficiency of the system was demonstrated by using a 3D electrode in a continuous flow reactor. The reduction of 3,4-dimethylmaleimides to the corresponding succinimides proceeds with a 3:2 diastereomeric ratio, which is independent of the nitrogen substituent and electrode surface area. The stereoselectivity of the process was rationalized by using DFT calculations, involving an acid-catalyzed tautomerization of a half-enol occurring through a double hydrogen-transfer mechanism.

## Introduction

Metal-catalyzed hydrogenation with H_2_ is widely employed for the reduction of C=C bonds to deliver the *syn* addition of H_2_ on the surface of a heterogeneous catalyst (e.g., Ni, Pd, Pt, Rh, Ru).[1] Although the process is 100 % atom-efficient, considerable process control is required to overcome mass transport limitations without engendering explosive hazards associated with the use of the highly flammable gas in pressurized reactors.[2] Conversely, the *anti* addition of H_2_ can be achieved only by using stoichiometric one-electron reductants to deliver electrons and protons to opposite faces of the alkene in two successive steps, typically by dissolving metals in a protic solvent (e.g., Na/NH_3_ or Mg/MeOH).[3] Apart from poor atom economy, such processes can also be difficult to scale up and produce metal-containing effluents.[4]

In comparison, electrochemical synthesis has the potential to deliver exceptional environmental and safety benefits. The need for stoichiometric and hazardous reductants is obviated by using electrons, water, and H^+^ as reagents, while electrodes can be regarded as heterogeneous catalysts that can be easily separated from the products and reused. Energy consumption is minimized by judicious reactor design and choice of electrode material; this allows reactions to occur under ambient conditions. Additionally, the chemoselectivity of an electrochemical reaction can be achieved, in part, by applying an electrode potential appropriate to the reduction of a particular functional group.

**Scheme 1:**
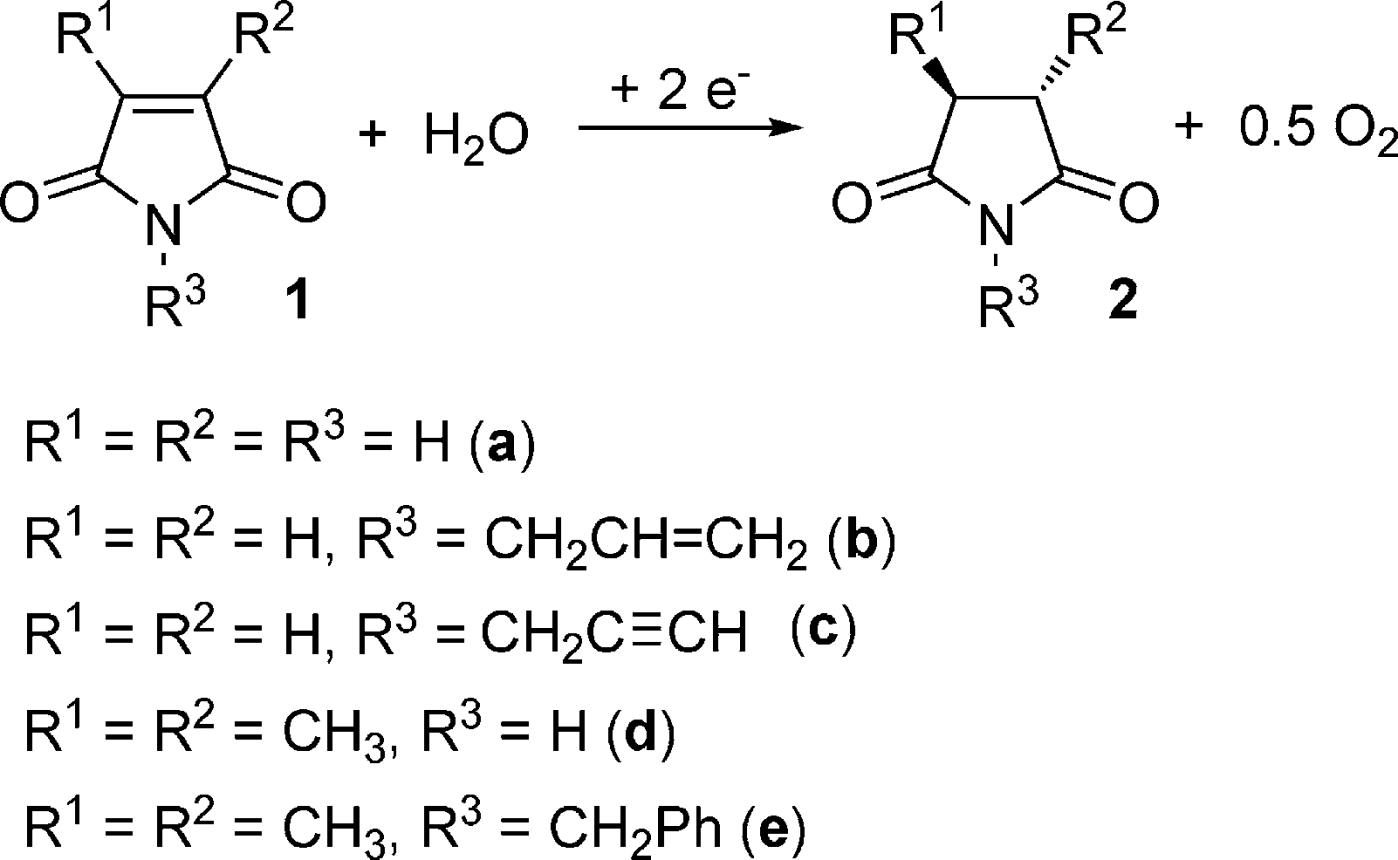
Electrochemical reduction of maleimides 1.

Recent advances in flow chemistry and the use of novel electrode materials and electrochemical reactors have greatly accelerated the development of electrosynthesis, overcoming limited productivity associated with mass transport limitations, as well as competitive reduction of the reaction solvent.[5] Herein, we describe the deployment of a laboratory-scale flow electrochemical reactor for the selective cathodic reduction of cyclic imides **1** to give succinimides **2** (Scheme 1).[6] The process affords clean and efficient reduction of the C=C bond under ambient conditions to generate O_2_ as the only byproduct.

## Results and Discussion

Electrochemical reduction of maleimide (**1 a**) to form succinimide (**2 a**) was first reported in 1937 to proceed at a lead or copper cathode in a solution of sulfuric acid containing elemental nickel particles in suspension.[7] Some four decades later, the reaction was re-examined by Barradas and co-workers, who reported the selectivity of the process to be highly pH-dependent: in a basic solution, maleimide undergoes hydrolysis and/or dimerization reactions,[8] whereas the reduction to succinimide occurs exclusively in acidic media.[9] These observations were similarly observed in a separate study of N-substituted maleimide compounds.[10] Although these studies focused on the mechanistic aspects of the reaction, the synthetic utility of the methodology had not been assessed adequately in terms of its productivity, its current efficiency/charge yield, as well as chemo- and stereoselectivity. Herein, we address these issues by studying the electrochemical reduction of five maleimide derivatives **1 a–e**. The corresponding succinimides of the methylated derivatives (**1 d** and **1 e**) are particularly interesting because they are reported to have antifungal activity against human opportunistic pathogenic fungi, including yeasts and dermatophytes.[11]

To eliminate possible metal-catalyzed hydrogenation resulting from surface hydride species and toxic metal residues, carbon-based electrodes, such as vitreous carbon (VC), graphite, and boron-doped diamond (BDD), were utilized during this work. The use of BDD is particularly attractive because it has a particularly high overpotential for hydrogen (and oxygen) evolution, offering a working electrode potential window of between about −0.75 and +2.35 V (vs. a standard hydrogen electrode (SHE)) in a 0.5 m aqueous solution of H_2_SO_4_.[12] Hence, loss in efficiency through competitive reduction rates of protons/water may be minimized, compared with rates on, for example, copper or lead.

**Figure 1:**
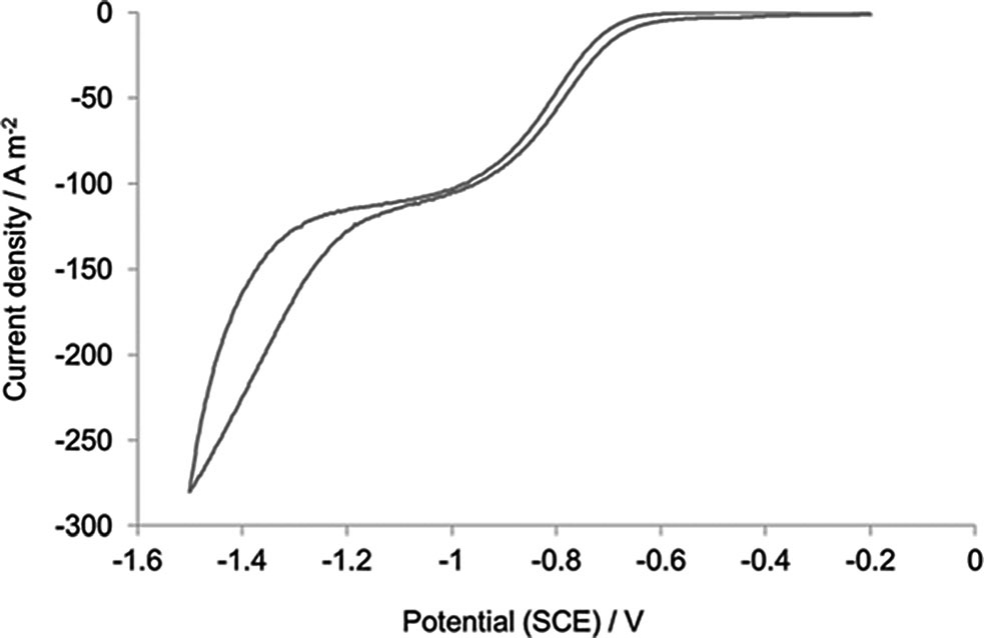
Cyclic voltammogram of 10 mm 1 a in 1 m H_2_SO_4_ at a VC RDE electrode. Scan rate 25 mV s^−1^; rotation rate=1000 rpm).

To identify the potential range over which reduction of **1 a** occurred under mass transport control, experiments were performed at a VC rotating disc electrode (RDE). With 10 mm of the substrate dissolved in 1 m H_2_SO_4_ (Figure 1), the onset of a two-electron reduction process was detected at −0.6 V (vs. SCE), with mass-transport-limited current densities achieved at potentials <−0.9 V (vs. SCE), below which the rate of H_2_ evolution increased significantly. At pH 2 and 4, the onset potentials for the reduction of the C=C bond occurred at slightly more negative values, and were accompanied by a decrease in current densities (Table 1, entries 2 and 3).

**Table 1:** Electrochemical characteristics of maleimide derivatives in an aqueous solution of H_2_SO_4_^[a]^

Compound	pH	Onset reduction potential vs. SCE [V]	Transport-limited current density^[b]^ [A m^−2^]
**1 a**	0	−0.9	130
	2	−1.1	60
	4	−1.0	45
**1 b**	0	−0.6	110
	2	−0.6	80
	4	−0.8	40
**1 c**	0	−0.6	80
	4	−0.8	40

[a] Recorded by using the VC RDE; 10 mm maleimide derivatives. See the Experimental Section for conditions. Standard reduction potentials are provided in Table S1 in the Supporting Information. [b] Two-electron reductions established by Levich plots (Figures S1 and S2 in the Supporting Information).

Subsequently, the onsets of the reduction of *N*-allyl (**1 b**) and *N*-propargyl (**1 c**) maleimides dissolved in 1 m H_2_SO_4_ and MeOH (4:1 *v*/*v*) were also determined to be −0.6 V at pH 0, but with diminished transport-limited current densities (Table 1); this suggested smaller diffusion coefficients for these N-substituted substrates. The effect of nitrogen substitution on the stability of radical intermediates has been reported previously.[10c] Crucially, no other reduction processes were detected within the potential range; thus, the chemoselective reduction of the electron-deficient alkene could be achieved in the presence of these other unsaturated moieties. In accordance with previous studies, increasing the pH of the solution decreased the onset electrode potentials (vs. SCE) for maleimide reduction and decreased the current densities. In contrast, no reduction waves could be recorded for the dimethyl-substituted maleimides **1 d** and **1 e**; the signals were obscured by significant competitive reduction of H^+^ to H_2_.

### Flow reactor

**Figure 2:**
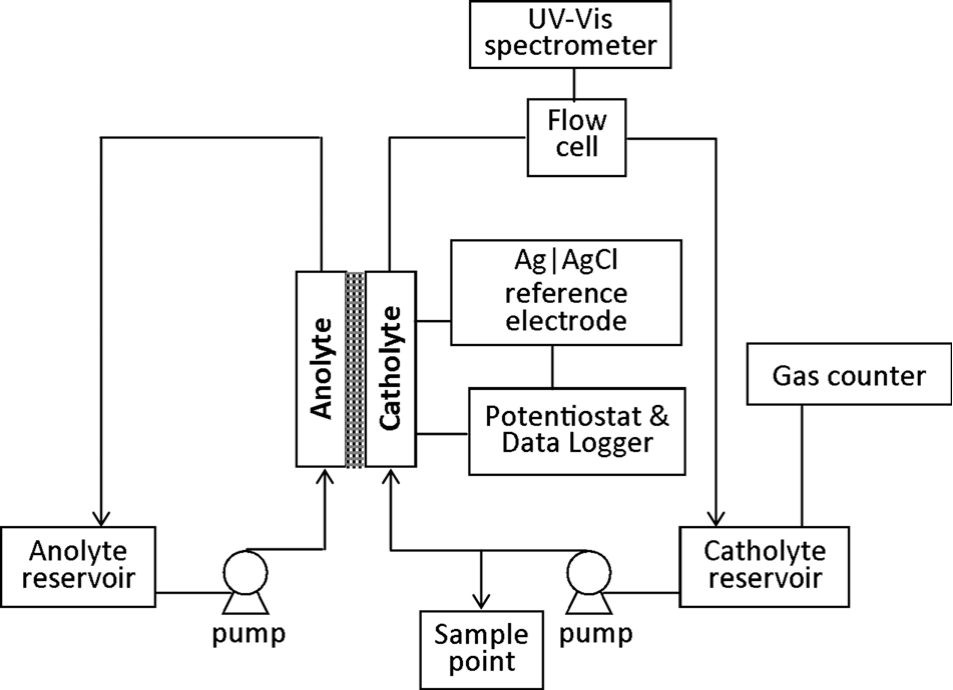
Schematic illustration of the electrochemical reactor operating in batch recycling mode described herein.

A commercial parallel-plate reactor (Electrocell microflow cell[13]) was modified for flow studies (Figure 2). Specifically, polytetrafluoroethylene (PTFE) gaskets were introduced to provide chemical inertness, prevent contamination, and to accommodate a AgCl|Ag reference electrode (see Figure 4 below in the Experimental Section). The flow reactor was operated in batch recycling mode with a continuously stirred tank reservoir. With the cathode controlled at a fixed electrode potential, current densities were monitored as a function of time. However, because maleimide reduction occurred in parallel with hydrogen evolution (*E*(H^+^/H_2_)≈−0.245 V vs. SCE), forming bubbles that adhered to the electrode, the effective area of the cathode/solution interface was altered, which caused time-dependence current densities as bubbles formed and detached. Hence, observation of the two different processes can be decoupled by the incorporation of a flow cuvette online to monitor maleimide formation through UV spectroscopy; a gas flow counter was installed at the cathode to monitor the competitive formation of H_2_. Product formation was independently verified by extracting an aliquot (1 mL) of the catholyte, which was neutralized to pH 10 with a 1 m aqueous solution of NaOH and extracted with dichloromethane. Following evaporation of the solvent, the residue was analyzed by ^1^H NMR spectroscopy.

Initially, experiments were performed with solutions of 10 mm of the maleimide substrate in 1 m H_2_SO_4_. This solution (100 mL) was recycled at a rate of 60 mL min^−1^ through the electrochemical reactor, with a potential of −1.2 V (AgCl|Ag) applied to the cathode. Reduction was counterbalanced by oxidation of water to generate O_2_ in the 1 m H_2_SO_4_ anolyte. By using a VC cathode, the reduction of **1 a** to **2 a** was detected, with 60 % conversion after 5 h, which corresponded to a charge yield of 62 % (Table 2, entry 1). In comparison, by using a BDD electrode,[14] 99 % conversion was obtained with a charge yield of 96 % (Table 2, entry 2) within the same time. This significant enhancement in performance can be attributed to the higher overpotential for hydrogen evolution at BDD compared with VC, so inhibiting the competitive reduction of protons to H_2_.

**Table 2:** Charge yields for the reduction processes using the flow reactor (2D electrode)^[a]^

Entry	Substrate	Cathode	*t* [h]	Conversion [%]^[b]^	Charge yield [%], 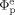 ^[c]^
1	**1 a**	VC	5	60	62
2	**1 a**	BDD	5	99	96
3	**1 b**	VC	5	79	79
4	**1 b**	BDD	5	>99	85
5	**1 c**	BDD	5	92	45
6	**1 d**	BDD	5	88	45
7	**1 e**	BDD	5	>99	41

[a] 10 mm of maleimide in 1 m H_2_SO_4_ (100 mL) was recirculated through the electrochemical reactor at 60 mL min^−1^ at room temperature. The applied potential of −1.2 V (AgCl|Ag). [b] Determined by ^1^H NMR spectroscopy, following neutralization with an aqueous solution of NaOH, and extraction with CH_2_Cl_2_. [c] Ratio of the theoretical charge (*Q*_p_) passed for product formation to the total charge (*Q*) passed during electrolysis.

The chemoselectivity of the reduction process was determined subsequently for substrates **1 b** and **1 c**, which contained *N*-allyl and -propargyl substituents, respectively. With a cathode potential of −1.2 V (vs. SCE), reduction of the electron-deficient cyclic olefin was achieved with complete chemoselectivity, at the expense of reduced charge yield (Table 2, entries 3–5). This may be attributed to slower rates of mass transport of these species to the cathode due to their larger molecular/hydrodynamic sizes, and hence, smaller diffusion coefficients.[15] The nature of the nitrogen substituent appeared to be particularly important in this context; charge yields of 85 and 45 % were recorded for **1 b** and **1 c**, respectively. This may be associated with the lower current densities recorded for these compounds (Table 1), although the correlation was nonlinear. The introduction of methyl substituents at C-3 and C-4 of **1 d** led to a further decrease in charge yields to 45 % (Table 2, entry 6), whereas the presence of both C and N substituents (**1 e**) did not appear to be additive (Table 2, entry 7). This was mainly attributed to the competitive reduction of protons at this applied potential. Nevertheless, in both cases, very high conversions to the corresponding succinimides were achieved in 5 h.

### Electrode geometry and productivity

The volumetric current density, and hence, conversion rate of the flow reactor was enhanced by increasing the specific surface area of the electrode. This was achieved by inserting an 11 mm thick graphite felt into the PTFE frame in contact with the BDD cathode. A low-resistance ohmic contact between a BDD plate feeder electrode and graphite felt enabled the active cathode area to be extended greatly (from 1.03×10^−4^ m^2^ to about 0.2 m^2^), which should enable the scale up of reaction rates. However, the local electrode potential is spatially distributed in 3D electrodes and varies with current density.[16]

In this experiment, the concentration depletion of maleimide was monitored by the disappearance of its UV absorption band at *λ*=276 nm. By using the 3D electrode, the conversion of maleimide was extremely rapid, so 95 % conversion was achieved in just 400 s, compared with 60 and 100 % conversions in 5 h for 2D VC and BDD electrodes, respectively. From an initial maleimide concentration of 0.1 m, the reaction was essentially complete within 2400 s, during which time 3645 C of charge was passed. Because the theoretical charge required for the two-electron reduction was 1929 C, this corresponded to a charge yield of about 50 %, but would have been greater at shorter times/lower conversions.

Raising the temperature to 50 °C increased the kinetics, with complete conversion of the maleimide to succinimide achieved in 300 s. This may be attributed to an increased diffusion coefficient for maleimide, and hence, increased transport-controlled current densities at the graphite cathode. However, it is worth noting that the range of potentials in such 3D electrodes is also sensitive to electrode and electrolyte conductivities, which are also temperature dependent.

### Chemoselectivity

**Scheme 2:**
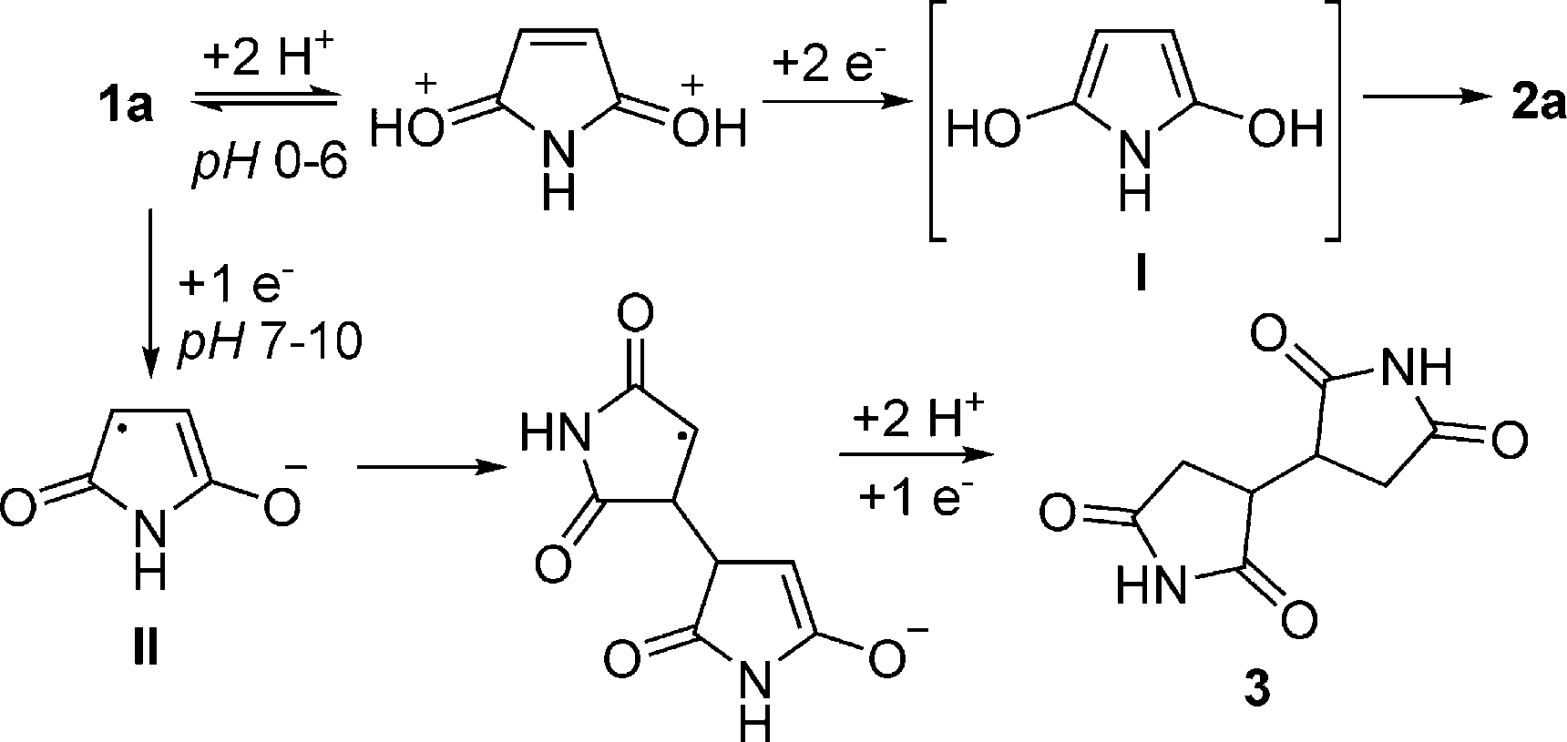
The pH-dependent chemoselectivity of the electrochemical reduction of 1 a.

Chemoselectivity of the electrochemical reduction of maleimide derivatives was reported to be highly dependent upon pH (Scheme 2).[8, 9] At pH values below 7, a two-electron reduction wave was detected; this resulted in the formation of the corresponding succinimides (**2**). At higher pH (7–10), stepwise one-electron reduction occurred, leading to dimerized products **3**. At even higher pH values (>10), hydrolysis of the maleimide becomes a competitive process.

The chemoselectivity of the reduction process towards different unsaturated carbon–carbon bonds was assessed by determining the reduction of several maleimide derivatives that contained *N*-allyl (**1 b**), -propargylic (**1 c**), and -benzyl (**1 e**) substituents (Table 3). In certain cases, the addition of methanol cosolvent was necessary to enable these compounds to dissolve in the electrolyte solution. As expected, dimerization of the maleimide was not detected under acidic conditions.[10c] More significantly, exclusive reduction of the cyclic C=C bond was attained at −1.2 V, whereas the N-substituents remained unaffected. This is particularly interesting because such orthogonal reactivity is difficult, if not impossible, to achieve by catalytic hydrogenation.

**Table 3:** Chemoselective reduction of maleimide derivatives by using a 3D graphite electrode^[a]^

Entry^[a]^	Compound	Applied potential [V]^[b]^	*t* [h]^[c]^	Conversion [%]^[d]^
1	**1 a**	−1.2	0.1	95
2	**1 b**	−1.1	0.5	92
3	**1 c**	−1.0	0.5	95
4	**1 d**	−1.2	0.5	94
5	**1 e**	−1.2	0.5	95

[a] General procedure: 10 mm of the maleimide substrate in 100 mL of 1 m H_2_SO_4_ or 1 m H_2_SO_4_/MeOH (4:1 *v*/*v*). Flow rate=60 mL min^−1^. [b] Value in V versus AgCl|Ag. [c] Monitored by UV/Vis spectroscopy. [d] Determined by ^1^H NMR spectroscopy.

### Stereoselectivity

The electrochemical reduction of *N*-ethyl-3,4-dimethylmaleimide was described briefly to produce both *cis* (*meso*) and *trans* (dl) diastereomers in approximately equal amounts at low pH.[10c] With this in mind, the stereoselectivity of this transformation was examined by comparing the reduction of 3,4-dimethyl-substituted maleimides **1 d** and **1 e** under different electrolysis conditions (Table 4). In all cases, a mixture of the diastereomers was obtained in a 2:3 ratio, with a slight preference for the *trans* isomer, that is, the product distribution was not dependent on either the electrode surface area (Table 4, entries 1 and 2) or the nitrogen substituent (Table 4, entry 3).

**Table 4:** Reduction of 3,4-dimethylmaleimides under different electrolysis conditions^[a]^

Entry	Compound	Electrode	Conversion^[b]^	*cis*/*trans*^[c]^
1	**1 d**	2D BDD^[d]^	99	2:3
2	**1 d**	3D graphite^[e]^	94	2:3
3	**1 e**	2D BDD^[d]^	99	2:3

[a] 10 mm substrate in 1 m H_2_SO_4_/MeOH (4:1 *v*/*v*; 100 mL). Potential of −1.2 V versus AgCl|Ag applied. [b] Determined by UV/Vis spectroscopy. [c] Determined by ^1^H NMR spectroscopy. [d] 5 h. [e] 30 min.

**Scheme 3:**
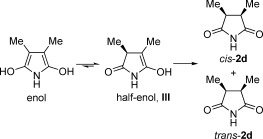
Stereodefining step in the reduction of 3,4-disubstituted maleimides.

It is generally accepted that the reduction proceeds through a CE (chemical–electrochemical) mechanism, in which two-electron transfer and the addition of H^+^ occur to generate the transient enol intermediate **I** (Scheme 2), which then undergoes tautomerization to form the succinimide. In the case of 3,4-disubstituted substrates (**1 d** and **1 e**), we postulated that the process occurred in a stepwise manner, such that the stereoselectivity was determined by the isomerization of a half-enol intermediate **III** (Scheme 3).

The enolization of *N*-methylsuccinimide under pH-neutral conditions was modeled previously by DFT computations using the B3LYP/6-31+G** basis set,[17] in which it was proposed that two water molecules were intimately involved in a triple hydrogen-atom transfer process. Herein, the model was adopted initially with slight modifications: by using the WB97XD/6-31G(d,p) basis set to account for dispersion, enhanced with a continuum solvation model (CPCM=water).

**Figure 3:**
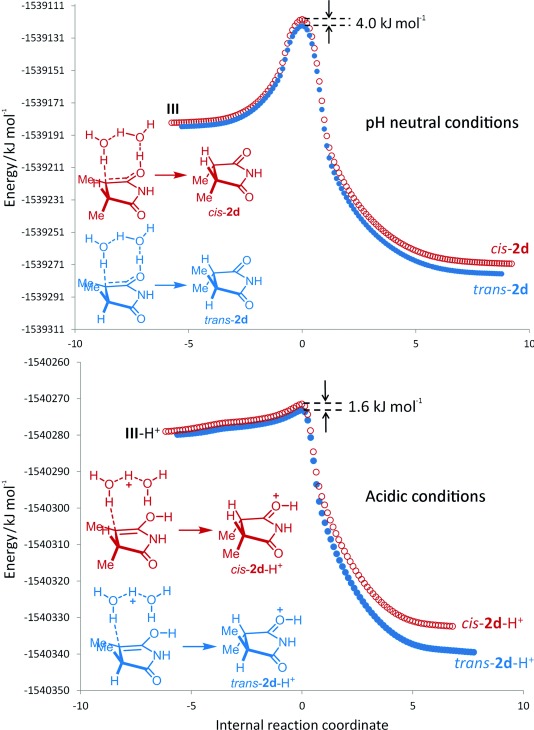
Computed energetic pathways for the tautomerization of half-enol to *cis*- (○) and *trans*-succinimide (•) under neutral, triple-hydrogen transfer (top) and acidic, double-hydrogen transfer (bottom) conditions.

Two sets of diastereotopic eight-membered cyclic transitions states were constructed for the half-enol **III** generated from substrate **1 d**, from which the energetic pathways leading to the *cis* and *trans* isomers could be plotted (Figure 3, top graph). In this model (triple hydrogen transfer), a 4 kJ mol^−1^ kinetic bias was observed for the *trans* isomer accompanied by a significant activation energy barrier; this does not agree with our experimental conditions/observations. However, by introducing a proton to the system, the overall energetic pathway was transformed greatly. In this case, only two hydrogen atoms were transferred (Figure 3, bottom graph). The spontaneity of the process is supported by a low activation energy barrier and, even more significantly, the formation of the *trans* isomer is predicted to be favored by 1.6 kJ mol^−1^; this corresponds to a selectivity of 1.9:1,[18] which may be considered to be in good agreement with the experimentally observed value. In theory, lowering the pH of the solution should increase the selectivity towards the *trans* isomer, but this is prohibited by the competitive dimerization process to form **3** (Scheme 2).

## Conclusions

We demonstrated that the reduction of the cyclic C=C bond in maleimide to form succinimide could be achieved exclusively in the presence of functional groups that were otherwise susceptible to competitive reduction under the conditions of catalytic hydrogenation. The reduction potential of these compounds was dependent on the nitrogen substituent, as well as on substituents at the C=C bond (Table 1).

The reactor-scale kinetics could be increased significantly by using a 3D graphite electrode (Table 3), although the competitive reduction of water was a problem in some of these systems (particularly **1 d** and **1 e**), in which there was a significant decrease in charge yield. We predict that 3D BDD electrodes would solve this issue; this will be investigated in our future work. The stereoselectivity for the reduction of 3,4-disubstituted maleimides is attributed to a solution process, through an acid-catalyzed tautomerization of a half-enol; the kinetic profile is commensurate with a double hydrogen atom transfer process. Preliminary theoretical calculations suggested that the selectivity was highly dependent on the nature of the C3 and C4 substituents; this will be studied in our future work.

## Experimental Section

### General

Commercial reagents were used as received. High-purity water was prepared by reverse osmosis and deionization. Solvents were dried over molecular sieve columns in a solvent purification system. Column chromatography was performed on flash silica gel (Kieselgel 60, 63–200 μm). The pH of solutions was measured by using a Hanna pH probe.

^1^H and ^13^C NMR spectra were obtained by using 400 MHz instruments (^1^H at 400 MHz and ^13^C at 100 MHz). Chemical shifts are reported in *δ* (ppm) and referenced to residual protons and ^13^C signals in deuterated chloroform. The coupling constants (*J*) are expressed in Hertz (Hz). Multiplicities are indicated as singlet (s), doublet (d), triplet (t), and multiplet (m).

IR spectra were recorded for solid or solution samples by using a PerkinElmer FTIR spectrometer fitted with an attenuated total reflectance (ATR) accessory. Mass spectra were recorded on a Micromass Autospec-Q mass spectrometer (EI and CI sources). Melting points (uncorrected) were determined by using an Electrothermal Gallenhamp apparatus and a calibrated thermometer (±2 °C). GC-MS results were recorded by using an HP GCD C GC-MS instrument and a Zebron Wax Plus column (Phenomenex).

HPLC was conducted by using a HP 1050 HPLC instrument and a Gemini NX C-18 column (Phenomenex). UV/Vis spectra were recorded by using an Agilent 8453 spectrophotometer, HELLMA fiber optic cables, and flow-through cuvette. Reaction progress was monitored by the disappearance of the absorbance band of the substrate at *λ*=276 nm.

To investigate the kinetics of the charge-transfer reactions, a VC RDE with a PTFE-sheathed 5 mm diameter (1.97×10^−5^ m^2^; Pine Instrument Company, USA) was used, and rotated at 3–50 s^−1^ by means of an AFMSRCE rotator controller (Pine Research Instruments, USA). A three-compartment glass cell was used for the rotating disc experiments that incorporated a vitreous disc electrode; a saturated calomel reference electrode (Monocrystally), which was assumed to have a potential of 0.254 V (SHE); and a platinum flag counter electrode.

A glass cell was used for batch electrolysis that incorporated a Nafion 117 (DuPont Inc.) cation-permeable membrane to separate the anolyte and cathode. A VC electrode (0.001 m^2^) was used as the cathode, a platinized titanium mesh as the anode, and a saturated calomel electrode (Monocrystally) as the reference electrode; the cathode was polished prior to each experiment. The anolyte and catholyte volumes were 270 mL; the former was a 1 m aqueous solution of H_2_SO_4_ and the latter was 0.01 m of the amide substrate with a 1 m aqueous solution of H_2_SO_4_ as the supporting electrolyte. High-purity nitrogen was bubbled through the catholyte to remove dissolved oxygen and to enhance mass transport rates.

An Autolab PGSTAT302N potentiostat (Metrohm Autolab) instrument was used to apply the required cathode potential and operated by using Nova software to monitor the resulting currents for experiments with the RDE and electrochemical reactor, typically at a range of scan rates (10 to 25 mV s^−1^).

**Figure 4:**
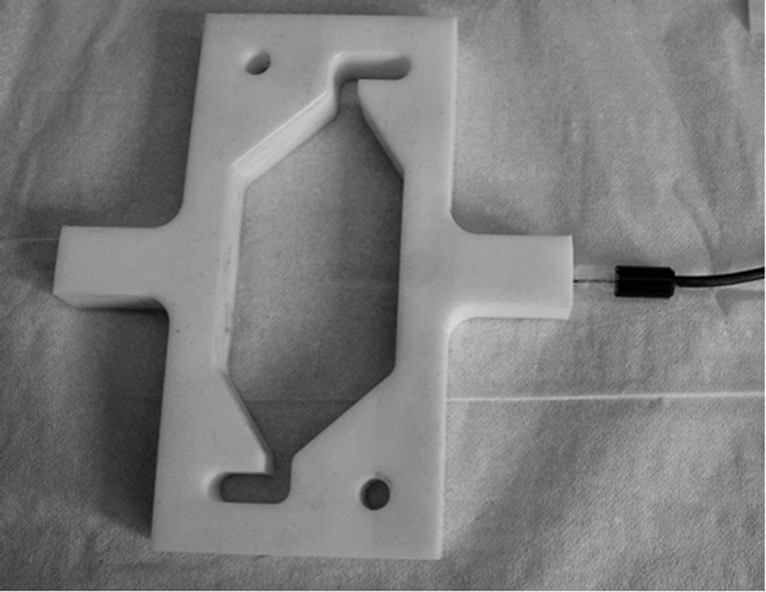
Modified catholyte chamber holding the Ag|AgCl reference electrode.

A commercial electrochemical reactor (Electrocell) was modified to accommodate a 10 mm thick 46×96 mm^2^ PTFE frame, which created a cathode compartment with a volume of 18.9 mL and an effective electrode geometric area of 103 mm^2^. An additional compartment was bolted onto the side of the reactor and designed to contain a AgCl|Ag wire reference electrode, which was connected hydraulically to the catholyte with a 0.5 mm hole drilled through the frame, and emerged close to the cathode surface (Figure 4). VC or BDD were used as the cathode materials, with Ti/Ta_2_O_5_–IrO_2_ as the anode because of its low overpotential for oxygen evolution and stability in acidic solutions. Nafion 117 ion-permeable membrane separated the anode and cathode compartments to prevent maleimides or their reduction products reaching the anode, at which otherwise they would have been oxidized. Hydrogen gas evolution rates were measured by using a Ritter MGC-1 gas counter.

### Preparation of 1 b by a Mitsunobu reaction[16, 19]

PPh_3_ (7.98 g, 30.6 mmol), maleimide (3.00 g, 31.2 mmol), and diisopropyl azodicarboxylate (DIAD; 6.9 g, 34.2 mmol) were added successively to a solution of allyl alcohol (1.65 g, 28.4 mmol) in THF (225 mL). The reaction mixture was left to stir overnight. The solvent was then evaporated and the product was isolated by column chromatography (5:1 hexane/EtOAc; *R*_f_=0.5) to give **1 b** as a yellow solid (1.72 g, 44 %). M.p. 41–43 °C (lit.[19] 40–42 °C); IR (ATR): 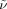
=3100, 3058, 2985, 1720, 1600, 1475, 1430, 1206, 900, 862, 635 cm^−1^; ^1^H NMR (400 MHz, CDCl_3_): *δ*=6.71 (s, 2 H), 5.82–5.72 (m, 1 H), 5.17–5.11 (m, 2 H), 4.12–4.06 ppm (m, 2 H); ^13^C NMR (100 MHz, CDCl_3_): *δ*=170.3, 134.2, 131.5, 117.6, 39.8 ppm; MS (EI): *m*/*z* (%): 138 (7) [*M*]^+^, 137 (100), 109 (15), 95 (23), 55 (40), 43 (78).

### General procedure for the preparation of maleimide derivatives 1 c–e

Propargylamine (1.69 g, 30.6 mmol), ammonium acetate (1.81 g, 23.4 mmol), or benzylamine (2.51 g, 23.4 mmol) were added to a solution of maleic anhydride or dimethylmaleic anhydride (2.0 g) in acetic acid (50 mL) and the reaction mixture was heated at reflux for 16 h. After cooling to room temperature, the solvent was evaporated and the product was purified by column chromatography.

*N*-Propargylmaleimide (**1 c**):[20] Yellow oil (0.53 g, 13 %); *R*_f_=0.4 (hexane/EtOAc, 7:3); IR (ATR): 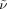
=3280, 3100, 2961, 1775, 1600, 1485, 1106, 805, 643 cm^−1^; ^1^H NMR (400 MHz, CDCl_3_): *δ*=6.73 (s, 2 H), 4.21 (d, *J*=2 Hz, 2 H), 5.17–5.1 ppm1 (t, *J*=2 Hz, 1 H); ^13^C NMR (100 MHz, CDCl_3_): *δ*=169.3, 134.5, 76.9, 71.6, 26.7 ppm; MS (EI): *m*/*z* (%): 136 (7) [*M*]^+^, 135 (82), 107 (100), 79 (30), 54 (75), 52 (48).

3,4-Dimethylmaleimide (**1 d**): White solid (1.62 g, 83 %); *R*_f_=0.4 (CH_2_Cl_2_); m.p. 109–112 °C (lit.[21] 111–113 °C); IR (ATR): 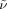
=3242, 1723, 1654, 1453, 1256, 1038, 904, 695, 650 cm^−1^; ^1^H NMR (400 MHz, CDCl_3_): *δ*=8.15 (s, 1 H), 1.96 ppm (s, 6 H); ^13^C NMR (100 MHz, CDCl_3_): *δ*=172.1, 138.3, 8.6 ppm; MS (EI): *m*/*z* (%): 126 (12) [*M*]^+^, 125 (100), 82 (10), 54 (68), 53 (30).

*N-*Benzyl-3,4-dimethylmaleimide (**1 e**): Yellow solid (2.23 g, 66 %); *R*_f_=0.4 (hexane/EtOAc, 7:3); m.p. 43–46 °C (lit.[22] 44–45 °C); IR (ATR): 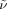
=3026, 1650, 1584, 1452, 1418, 1390, 1035, 963, 706, 650 cm^−1^; ^1^H NMR (400 MHz, CDCl_3_): *δ*=7.25–7.35 (m, 5 H), 4.66 (s, 2 H), 1.97 ppm (s, 6 H); ^13^C NMR (100 MHz, CDCl_3_): *δ*=171.8, 137.3, 136.7, 129.3, 128.6, 128.4, 127.7, 41.5, 8.7 ppm; MS (EI): *m*/*z* (%): 217 (2) [*M*]^+^, 216 (25), 215 (100), 187 (28), 172 (35), 104 (35), 91 (32), 77 (15), 54 (17).

### Electrochemistry

The electrochemical cell was operated in batch recycling mode by using a Masterflex peristaltic pump (Model 07524-40) to supply flow rates of up to 90 mL min^−1^ through the catholyte compartment. Both electrode compartments were filled with 1 m H_2_SO_4_ unless otherwise stated. The glass catholyte and anolyte reservoirs had a volume of 100 mL each.

At time *t*=0, recirculation of both cell compartments was established, the operating potential was applied and the resulting current was monitored continuously. Experiments were concluded after a maximum of 5 h, depending on the progress of the reduction process. The catholyte was neutralized by the addition of 1 m NaOH, and extracted with dichloromethane. The combined organic extracts were dried over MgSO_4_ and the solvent was evaporated. The residue was analyzed by NMR spectroscopy.

The NMR spectroscopy data for **2 a**,[23] **2 b**,[24] and **2 c**[25] are consistent with values reported in the literature.

Compounds **2 d** and **2 e** were obtained as a mixture of diastereoisomers

3,4-Dimethylsuccinimide **2 d**: *cis* isomer:[26] ^1^H NMR (400 MHz, CDCl_3_): *δ*=8.52 (br s, 1 H), 3.00–2.90 (m, 2 H), 1.18 ppm (d, *J*=7.0 Hz, 6 H); *trans* isomer:[27] ^1^H NMR (400 MHz, CDCl_3_): *δ*=9.35 (br s, 1 H), 2.49–2.41 (m, 2 H), 1.31 ppm (d, *J*=7.0 Hz, 6 H).

*N*-Benzyl-3,4-dimethylsuccinimide **2 e**: *cis* isomer:[28] ^1^H NMR (400 MHz, CDCl_3_): *δ*=7.34–7.21 (m, 5 H), 4.59 (s, 2 H), 2.99–2.91 (m, 2 H), 1.16 ppm (d, *J*=7.0 Hz, 6 H); *trans*-isomer:[6b] ^1^H NMR (400 MHz, CDCl_3_): *δ*=7.34–7.21 (m, 5 H), 4.59 (s, 2 H), 2.49–2.40 (m, 2 H), 1.29 ppm (d, *J*=7.0 Hz, 6 H).

### DFT calculations

DFT calculations were performed by using the Gaussian 09 software package.[29] Reactants and products were optimized by using the WB97XD/6-31G(d,p) level of theory. Transition states were optimized by using either QST2 or the Berny algorithm, which was frequency checked, followed by intrinsic reaction coordinate (IRC) calculations. Output files have been deposited on a server and are available through the following links: 1) *cis*-succinimide (neutral): http://dx.doi.org/10.6084/m9.figshare.928138; 2) *trans-*succinimide (neutral): http://dx.doi.org/10.6084/m9.figshare.928139; 3) *cis*-succinimide (acid catalyzed): http://hdl.handle.net/10042/132753; 4) *trans-*succinimide (acid catalyzed): http://hdl.handle.net/10042/132754

## Supporting Information

As a service to our authors and readers, this journal provides supporting information supplied by the authors. Such materials are peer reviewed and may be re-organized for online delivery, but are not copy-edited or typeset. Technical support issues arising from supporting information (other than missing files) should be addressed to the authors.

miscellaneous_information
