# Supplementary material for: Chemo- and Diastereoselectivities in the Electrochemical Reduction of Maleimides
Source: ChemSusChem. 2015 Jan 8;8(4):665–71. doi: 10.1002/cssc.201403184 (PMC4498473; doi:10.1002/cssc.201403184)
Supplement: Supplementary file 1 — miscellaneous_information [file cssc0008-0665-sd1.pdf]

CHEMISTRY & SUSTAINABILITY
CHEMSUSCHEM
ENERGY & MATERIALS

Supporting Information

© Copyright Wiley-VCH Verlag GmbH & Co. KGaA, 69451 Weinheim, 2015

Chemo- and Diastereoselectivities in the Electrochemical Reduction of Maleimides

Kathryn Rix,[a] Geoffrey H. Kelsall,[b] Klaus Hellgardt,[b] and King Kuok (Mimi) Hii*[a]

cssc_201403184_sm_miscellaneous_information.pdf

Supporting Information

Chemo- and Diastereo-selectivities in the Electrochemical Reduction of Maleimides

Kathryn Rix,[a] Geoffrey H. Kelsall,[b] Klaus Hellgardt,[b] and King Kuok (Mimi) Hii*[a]

Mass transport and Levich plot

Figure shows the cyclic voltammogram (CV) for the reduction of 0.01 M maleimide **1a** in 1 M aq. H_2SO_4 using a Pb-coated VC RDE; the potential was scanned between -0.4 and -1.4 V (SCE) at a scan rate of 25 mV s^{-1} and at a range of rotation frequencies. The CV curves show plateau regions around -1.2 V, suggesting that the reduction process is operating under mass transport control.

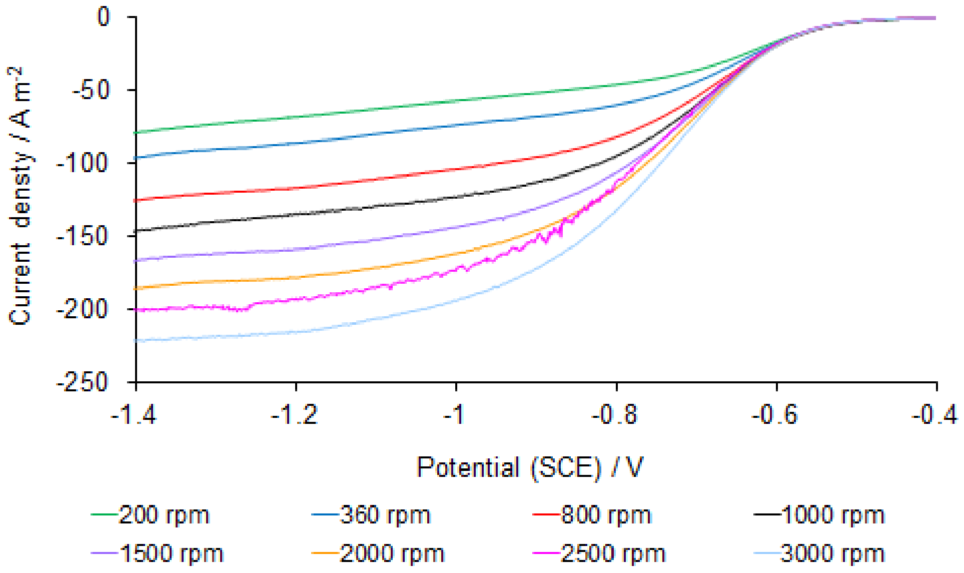

Figure S1. Cyclic voltammogram for the reduction of 0.01 M maleimide **1a** in 1 M aq. H_2SO_4 at a Pb-coated VC RDE; electrode potential swept between -0.4 V and -1.4 V (SCE) at a scan rate of 25 mV s^{-1} and at a range of rotation rates.

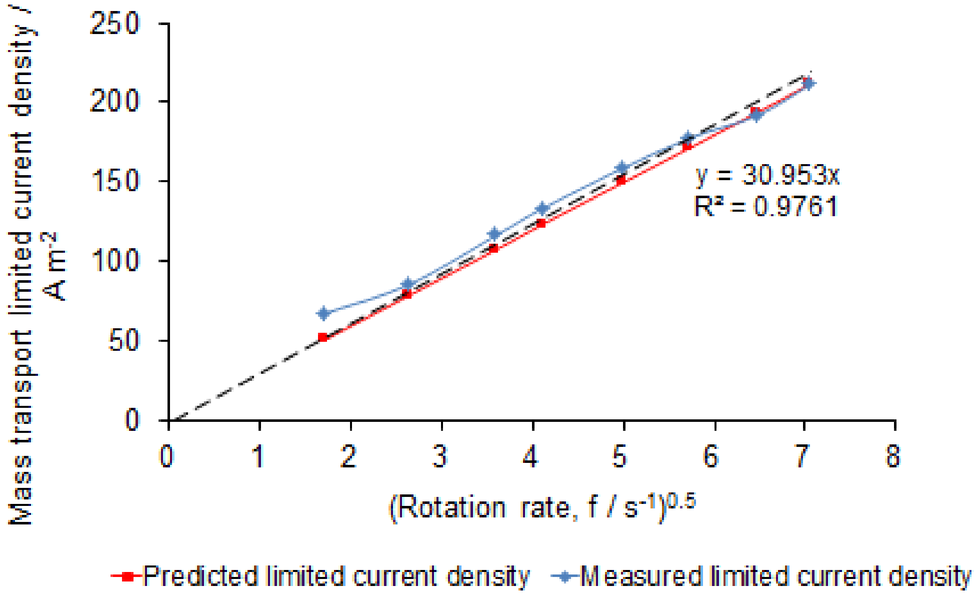

Figure S2. Effect of rotation rate on measured current densities for the reduction of 0.01 M maleimide **1a** in 1 M aq. H_2SO_4 using a Pb-coated VC RDE compared with those predicted by Levich's equation.

As the electron transfer reaction is mass transport controlled, the limiting current density is given by the Levich equation:

$$j_L = -1.554 nFD^{2/3}\omega^{1/2}\nu^{-1/6}c$$

A linear Levich plot (square root of the rotation frequency against the limited current density, Fig. S2) was observed, confirming that the reduction process was mass transport controlled. The limiting current density for maleimide **1a** was calculated using Levich equation, when n = 2, D = 1×10^{-9} m^2 s^{-1} and c = 10 mol m^{-3}. The plot shows a linear trend line can be fitted to the measured data which passes through the origin, thus establishing a 2-electron reduction process.

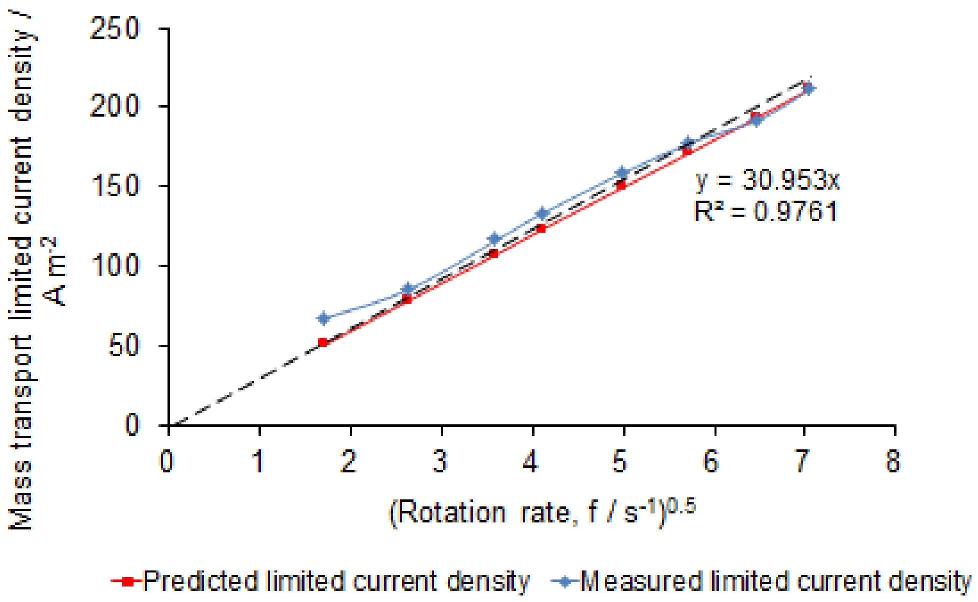

Figure S2. Effect of rotation rate on measured current densities for the reduction of 0.01 M maleimide **1a** in 1 M aq. H_2SO_4 using a Pb-coated VC RDE compared with those predicted by Levich's equation.

Table S1. Standard potentials for the reduction of **1a-c** vs SHE.

Compound	Standard potentials vs SHE/V
**1a**	-0.12
**1b**	-0.19
**1c**	-0.18
